# Early Complications Following Articular Calcaneus Fracture Repair: Evaluation of Open Versus Percutaneous Techniques

**DOI:** 10.1097/OI9.0000000000000049

**Published:** 2019-12-20

**Authors:** Derrick M. Knapik, Michael J. Hermelin, Joseph E. Tanenbaum, Heather A. Vallier

**Affiliations:** Department of Orthopaedic Surgery, MetroHealth Medical Center, Affiliated with Case Western Reserve University, Cleveland, OH.

**Keywords:** calcaneus fracture, complications, minimally invasive, percutaneous

## Abstract

**Objectives:**

To assess complications and secondary operations in patients treated with either open reduction and internal fixation (ORIF) versus percutaneous fixation of displaced intra-articular calcaneus fractures.

**Design:**

Retrospective comparative study.

**Setting:**

Level 1 trauma center.

**Patients/Participants:**

Ninety-three adult patients with 111 fractures treated by a single orthopaedic traumatologist between 2001 and 2014.

**Intervention:**

ORIF through an extensile lateral approach or percutaneous reduction and internal fixation.

**Main Outcome Measurements:**

Wound-healing complications, infections, posttraumatic arthrosis (PTOA), and secondary procedures.

**Results:**

Fifty patients with 58 fractures underwent ORIF, and 43 patients with 53 fractures had percutaneous fixation. Mean age was 43 years, and 80% were male. Open fractures and two-part fractures were more often treated percutaneously (26% vs 8%, *P* = 0.03) and (49% vs 31%, *P* = 0.02), respectively. Patients undergoing percutaneous fixation were more often tobacco users (58% vs 36%, *P* = 0.04) and with history of alcohol and other substance abuse. Twenty-seven patients (29%) had 28 complications, including 21% with PTOA, with no differences based on type of treatment. Six patients had secondary procedures, with no difference based on type of treatment. Patients with open fractures (*P* = 0.001) or tobacco abuse (*P* = 0.005) were more likely to experience complications.

**Conclusions:**

No differences in complication rates were found for ORIF versus percutaneous fixation. Regardless of fixation technique, patients with open fractures or history of tobacco abuse were more likely to develop complications. Percutaneous reduction and fixation represents an alternative to extensile ORIF in terms of similar early and late complications, particularly in high risk patients.

**Level of Evidence:**

Therapeutic Level III

## 1 Introduction

Calcaneus fractures account for 1% to 2% of all fractures and 75% are articular.^[1]^ Displaced fractures are associated with a high degree of morbidity and long-term dysfunction.^[2]^ Controversy persists regarding optimal management of articular calcaneal fractures, with most studies demonstrating no significant differences in complication rates at short or long-term follow-up after comparing operative and nonoperative management.^[2,3–7]^ However, multiple recent investigations have demonstrated superior outcomes in select patients with displaced articular fractures treated surgically.^[8–14]^

Over the last 3 decades, ORIF via the extensile lateral approach has become the most common approach for the treatment of intra-articular calcaneal fractures.^[4,7–9]^ While ORIF permits optimal visualization of the subtalar joint and facilitates accurate joint reduction,^[5,6]^ frequent infections and wound-healing complications have been documented.^[4,10,13,15–20]^ These complications have been shown to occur predominately in patients with such comorbidities as smoking, diabetes, vascular disease, and in those with severe soft tissue trauma.^[7]^ Less invasive techniques have been introduced to minimize complications associated with open treatment, especially in high-risk surgical patients.^[21,22]^ Percutaneous reduction and fixation is a safe alternative with low complication rates.^[5,20,21,23–27]^ However, few studies to date have compared outcomes for articular calcaneal fractures treated with percutaneous reduction and fixation versus ORIF.^[9,25]^

The purpose of this study was to review articular calcaneus fractures treated with either percutaneous fixation or ORIF via the extended lateral approach. Specifically, comparison of fixation techniques was based on early and late complications and reoperations. Analysis of patient comorbidities and fracture characteristics was undertaken to assess for factors predictive of complications. The authors hypothesized percutaneous patients may have more risk factors for complications, while patients treated percutaneously would have fewer complications.

## 2 Patients and Methods

All patients sustaining a displaced intra-articular calcaneal fracture (AO/OTA 82C)^[28]^ treated at a Level I trauma center by a single fellowship trained orthopaedic traumatologist between September 2001 and June 2014 were included. Fractures were classified as nondisplaced (82-C1), two-part (82-C2), three-part (82-C3), or four or more parts (82-C4). Fractures were also classified as described by Sanders (types 2, 3, or 4).^[12]^ Patients underwent fixation using one of 2 treatment modalities: ORIF or percutaneous reduction and fixation. Those treated nonoperatively were excluded. Open and closed industrial injuries causing crushing of the foot from heavy weight, and resulting in multiple foot fractures were also excluded. Patients with adjacent injuries of the ankle and distal tibia were also excluded.

Choice of treatment was at surgeon discretion. While establishing accuracy of hindfoot and articular alignment was a high priority, the risks of soft tissue injury and patient medical and social factors, along with simplicity of fracture pattern, also influenced treatment recommendations. Open reduction and internal fixation was undertaken within several days after injury, once soft tissue swelling was deemed to be improved. An extensile lateral approach was performed. Fractures were reduced and stabilized with mini-fragment and small fragment standard stainless steel plates and screws, depending on the fracture pattern. Allograft bone was applied to subchondral and tuberosity defects in all patients undergoing ORIF. Intraoperative fluoroscopy was utilized to assess reduction quality. Occasionally plain radiographs were obtained in the operating room. Closure was performed over a suction drain.

Percutaneous reduction was performed through small stab incisions with Schanz pins, elevators, and clamps to restore height and congruity of the posterior facet and to improve calcaneal length and width. Instruments and implants were placed through the tuberosity through various lateral and/or posterior locations as needed. Formal exposure to visualize reductions was not performed. Depending on the fracture pattern and bone quality, up to several solid, small fragment, stainless steel screws were inserted to maintain reduction. For comminuted fractures, occasionally Kirschner wires spanning the subtalar joint provided additional fixation over the first several weeks. Ends of wires were left outside the skin and were later removed in the outpatient clinic. Open fractures were treated with urgent surgical debridement, with staged fixation depending on the soft tissue quality and systemic status of the patient. For patients with bilateral calcaneal fractures in which one of the fractures was managed nonoperatively (n = 4), only the operatively treated fracture was included, classifying these patients as a single fracture.

Demographic data, employment status, medical comorbidities including diabetes mellitus, hypertension, substance abuse and psychiatric history; and injury and treatment features were documented. Complications were recorded, including superficial infection (surgical wound erythema or wound dehiscence treated with local wound care and oral antibiotics) and deep infection (surgical wound erythema or dehiscence with purulent drainage treated with surgical debridement and intravenous antibiotics). Fracture union was assessed with serial radiography obtained at approximately 6 to 8-week intervals after surgery. Postoperative reduction quality was defined on plain radiography by restoration of Bohler's angle and the angle of Gissane, compared with the uninjured foot. Reduction of the posterior facet was assessed for residual displacement (anatomic: 0–1 millimeters (mm) of malalignment, near anatomic: 2–3 mm, and approximate: 4–5 mm).^[11]^ Postoperative CT scans were not obtained. Posttraumatic osteoarthrosis (PTOA) was defined radiographically after minimum 12 months as new or progressive (compared with preinjury) decrease in subtalar joint space with development of osteophytes, subchondral cysts, and/or sclerosis on plain radiography. Secondary procedures included any unanticipated procedure, including implant removal, subtalar arthrodesis, and amputation. Data were collected by trained providers not involved in the care of the patients.

### 2.1 Statistical analysis

Continuous variables were compared using the Student *t* test and categorical variables compared using the Chi-square test. A Fischer exact test was performed in cases in which a variable had an expected frequency of 5 or less. A *P* value of < .05 was used to determine statistical significance. All statistical analyses were performed using SPSS (Version 23, IBM, Armonk, NY) software.

## 3 Results

A total of 93 patients sustaining 111 displaced intra-articular calcaneal fractures were managed operatively by the senior author. They had mean age at the time of surgery of 43.1 years [range: 21–79] and were 80% males (Table 1). A total of 72% (n = 67) of patients were employed prior to injury. The most common mechanism of injury was a fall from height (n = 58, 62%), followed by motor vehicle collisions (n = 34, 37%). Bilateral fractures were present in 19% (n = 18), of which 4 patients were treated nonoperatively on 1 side. Sixteen percent (n = 15) sustained open fractures, all type 3A. Additional injuries were present in 66% of patients. Patient comorbidities at the time of injury consisted of: tobacco smoking (n = 44, 47%), psychiatric history (n = 14, 17%), hypertension (n = 12, 13%), recreational drug use (n = 12, 13%), alcohol abuse (n = 11, 12%), and diabetes mellitus (n = 6, 6%). Seven patients (7.5%) had work-related injuries and associated workers’ compensation claims.

**Table 1:**
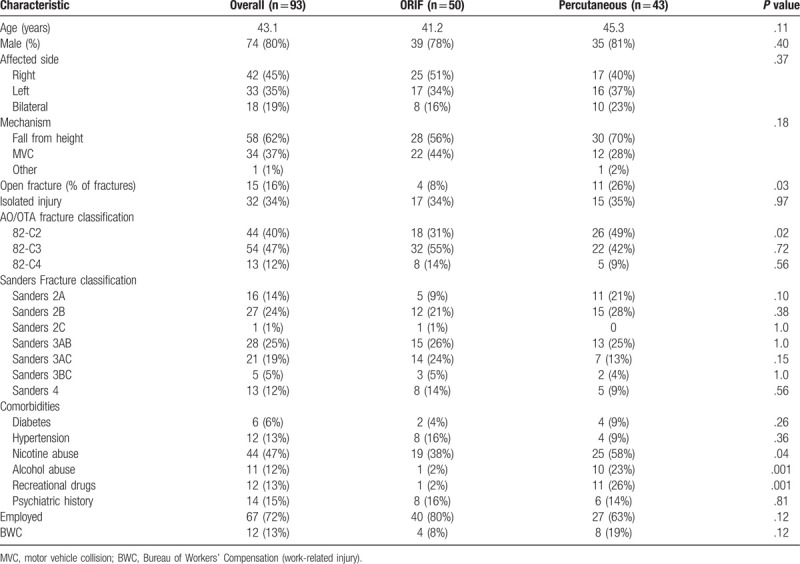
Demographic and injury characteristics of patients treated with ORIF or percutaneous techniques.

**Figure 1:**
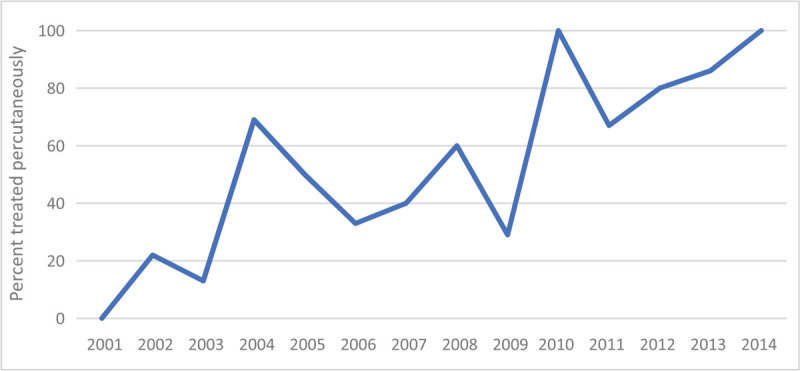
The number of fractures treated percutaneously increased over the period of study. The percent of fractures treated percutaneously is shown for each year of the study.

Open reduction and internal fixation was performed in 54% (50 patients with 58 fractures), and percutaneous fixation in 46% (43 patients with 53 fractures) (Table 1). The number of fractures treated percutaneously increased over the period of study (Fig. 1). Time from injury to surgery was significantly longer in patients undergoing ORIF (mean, 9.1 days (range 2–24)) compared with percutaneous fixation (mean, 4.6 days (range 0–22); *P* < .001). Patients with fractures classified as 82-C2 or Sanders type 2 fractures were more often treated with percutaneous fixation than with ORIF (*P* = .02), and open fractures were more likely to be managed percutaneously (11 of 15 open fractures (73%, *P* = .03)). All open C2 fractures were treated percutaneously, and half of these patients were tobacco smokers. Overall, patients undergoing percutaneous fixation were more likely to be smokers (58% vs 38%, *P* = .04) and to have history of alcohol abuse (23% vs 2%, *P* = .001), or history of recreational drug use (26% vs 2%, *P* = .001). Other medical and social conditions and injury features were not associated with treatment type.

**Figure 2:**
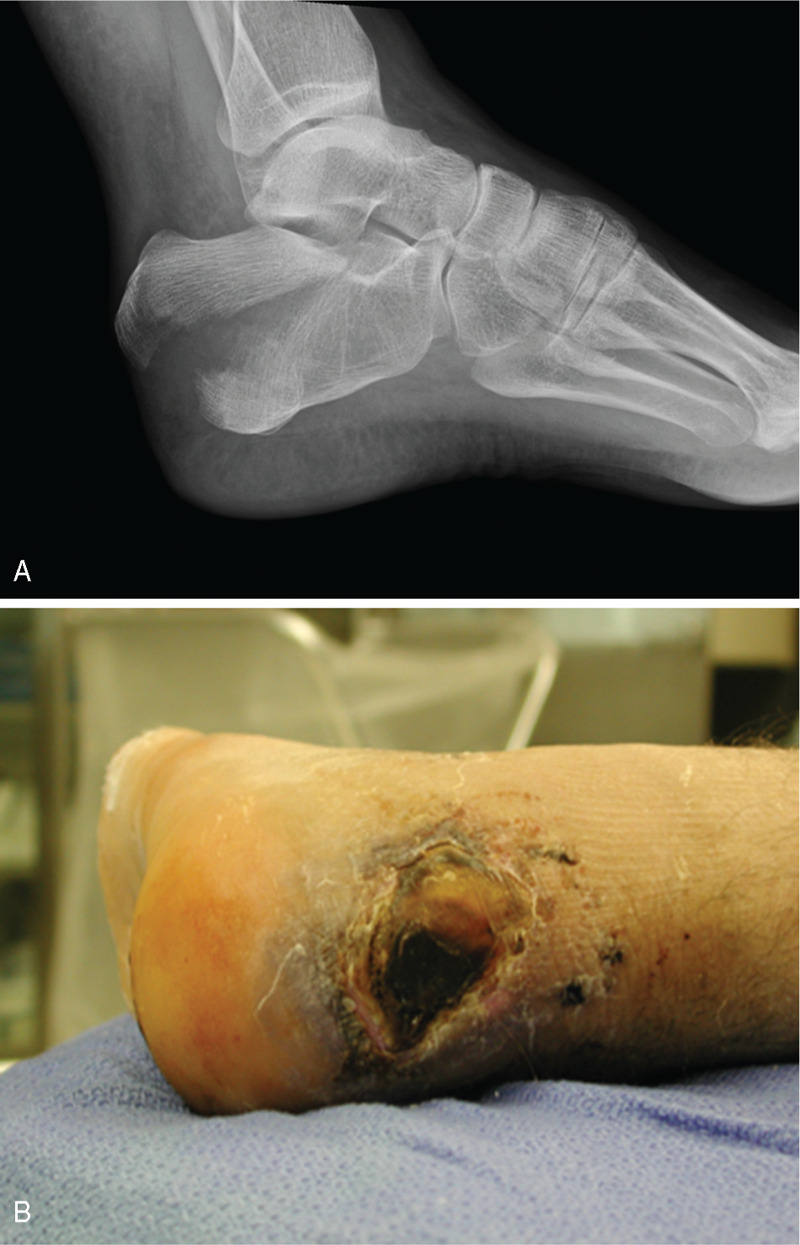
A 22-year-old man fell from a height, sustaining a displaced tongue type fracture (A: lateral foot radiograph). He was initially evaluated at an outside hospital where he was given instructions to return. He presented to our clinic 8 days later with necrosis of the skin surrounding the fracture (B: clinical photograph). Despite urgent reduction and fixation, with local wound debridement and coverage, he developed osteomyelitis.

After mean follow-up of 47 months (range, 3–171 mo) 93 patients were clinically evaluated. A total of 27 patients with 28 postsurgical complications were identified (Table 2). This included 16 patients with 17 complications after ORIF and 11 patients with 11 complications after percutaneous fixation. Precautionary oral antibiotics at the discretion of the treating surgeon were given to 4 patients after ORIF and 5 patients after percutaneous treatment for postoperative swelling and erythema, with or without serous drainage. Three of these patients had a few millimeters of epidermolysis along the corner of the wound after ORIF, which was treated with alcohol, nonadherent dressing, and splintage. All resolved over a period of several weeks. One patient presented to our outpatient clinic several days after he sustained a closed displaced, intra-articular, tongue type fracture. At the time of presentation necrosis of the skin surrounding the displaced tongue fragment was noted (Fig. 2). He underwent urgent percutaneous reduction and fixation of the fracture. He experienced further hindfoot necrosis and deep infection, treated with serial debridements and attempted rotational tissue coverage. His osteomyelitis persisted, and he ultimately underwent transtibial amputation.

**Table 2:**
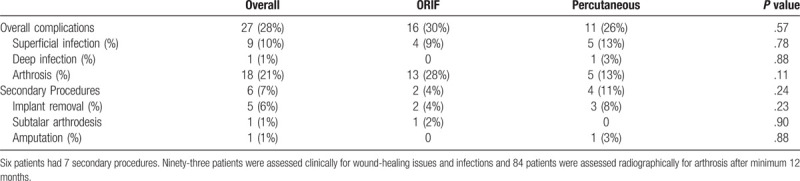
Complications and secondary operations following ORIF versus percutaneous fixation.

Plain radiography was evaluated to assess quality of reduction. Bohler's angle was a mean of 30 degrees (range 27–41 degrees), postoperatively. This measurement was considered completely restored in all patients after ORIF and in 46 fractures (85%) treated percutaneously (*P* = .005), and within 5 degrees of normal in the other 15%. The angle of Gissane was considered restored in all patients after ORIF and in 44 fractures (83%) treated percutaneously (*P* = .009). Reduction quality of the posterior facet after ORIF was anatomic in 88% of patients after ORIF, which was more often than with percutaneous reduction (38%, *P* = 0001). Near anatomic reductions were noted in 12% after ORIF, while percutaneous reduction yielded 51% near anatomic and 11% approximate reductions.

All fractures united primarily. After minimum follow-up of 12 months (mean 51 months; range, 12–171), 84 of 93 patients were evaluated with radiography. This included 46 of 50 (92%) treated with ORIF and 38 of 43 (88%) after percutaneous fixation. Eighteen fractures (21%) developed PTOA, with a trend for it to be more likely after ORIF (28% vs 13%, *P* = .11). Of those with PTOA after ORIF, 62% had an anatomic reduction, versus 20% after percutaneous treatment; PTOA was more likely after nonanatomic reduction (*P* = .006). With the numbers of patients available we were not able to identify associations between fracture pattern, comminution, or presence of open fracture and the development of PTOA. Implant removal was performed due to prominent implants causing irritation in 5 patients (n = 2 ORIF, n = 3 percutaneous). No significant differences in complication rates (*P* = .57) or rates of secondary procedures (*P* = .24) were appreciated based on fixation approach. Patients with open fractures (*P* = .001) and those with a history of tobacco smoking (*P* = .005) were more likely to develop a complication. Complications were not related to fracture pattern, workers compensation status, diabetes, hypertension, alcohol abuse, or recreational drug use.

## 4 Discussion

No significant differences in overall complication rates were appreciated between patients treated with ORIF versus percutaneous fixation for intra-articular calcaneal fractures. Those treated with percutaneous fixation were more often a simple pattern but were also more often open fractures. Percutaneously treated patients were more likely to have a history of nicotine, alcohol, and/or recreational drug abuse. Regardless of fixation type, patients with open fractures and those with a history of tobacco smoking were more likely to experience a complication.

The lack of a significant difference in complications and secondary procedures between patients undergoing ORIF versus percutaneous fixation supports the findings from De Boer et al.^[9]^ The authors reported on 78 patients with articular calcaneal fractures undergoing ORIF (n = 27) or percutaneous fixation (n = 33) and found no significant difference in overall complications after ORIF (16%) versus percutaneous fixation (13%).^[9]^ However, their patients treated percutaneously had a higher risk for requiring late interventions (subtalar arthrodesis, exostosis resection, wound debridement, revision surgery), specifically subtalar arthrodesis and implant removal. Examination of individual complications in our study demonstrated no differences between treatment techniques. Another comparative study reported on 128 patients with 125 fractures treated with either ORIF (n = 41) or percutaneous fixation (n = 79), noting patients undergoing ORIF had more deep infection and minor wound complications when compared with those treated percutaneously.^[25]^

The frequency of wound-healing complications and of subtalar arthrosis following ORIF reported in this study are within the range of previously reported complication rates for articular calcaneal fractures, reported between 0.4% and 27%.^[4,29]^ Moreover, our complication rate following percutaneous fixation is also within previously reported ranges of 2.4% to 46%.^[9,24,30,31]^ Soft tissue complications have been highly variable in prior literature, and in percutaneously treated patients may be attributed to more open fractures and to higher rates of nicotine, alcohol, and illicit substance abuse. These patient and injury features are well known to contribute to poor wound healing and poor adherence to physician instructions following discharge.^[16,17,32]^ However, the minimally invasive technique afforded by percutaneous fixation is performed by the senior author for socially complicated patients with pre-existing medical comorbidities due to the high risk of poor soft tissue healing. This tactic has potential for producing better alignment and better function when compared with nonoperative management in these high-risk patients.^[9]^ It is possible that a sinus tarsi approach with better articular visualization though limited soft tissue dissection may afford the best of both worlds in terms of accuracy of reduction with low rates of wound-healing issues and infections.^[33–39]^

Secondary procedures primarily involved implant removal, which was performed in 6% of all patients, unrelated to technique of treatment. This is lower than rates of secondary procedure reported in other investigations. De Boer et al^[9]^ reported implant removal in 39% of patients treated with ORIF and 66% with CRPP. No patient who underwent ORIF required subtalar arthrodesis, while 20% of patients undergoing CRPP had subtalar arthrodesis, comparable to the 15% rate reported by Schepers et al.^[30]^ We noted better quality of reduction in patients after ORIF; however, some patients in both treatment groups developed arthritis, even when the reduction was considered anatomic. This is likely related to initial injury severity. In Sanders et al long-term investigation of Sanders type II and type III fractures (minimum of 10 years follow-up), subtalar arthrodesis was performed in 29% following ORIF, with type III fractures being 4 times at higher risk versus type II.^[7]^

Regardless of fixation technique, patients with a history of nicotine dependence and open fractures were at more risk for development of a complication. In their study of 170 patients with 190 calcaneal fractures, Folk et al^[17]^ found that patients were at higher risk for wound infections if they sustained open fractures or reported a history of smoking. Other authors described ORIF via the extensile lateral approach in 341 closed and 39 open fractures, and found serious infections in 1.8% of closed compared with 7.7% of open fractures, all of which required treatment beyond antibiotics.^[16]^ Furthermore, Assous and Bhamra^[32]^ found a significantly higher rate of wound infections in smokers following internal fixation (70% versus 15%), consistent with reports of longer time for wound healing in smokers following ORIF.^[33]^

Fractures involving two fragments were more likely to be repaired percutaneously than with ORIF. This is reasonable to expect, as current literature suggests that percutaneous fixation for 2-fragment injuries is associated with good results, likely secondary to less injury complexity.^[22,25,33,40]^ Due to the minimally invasive nature of percutaneous fixation, surgical intervention may be safely performed within several days following injury compared with ORIF, where treatment may be delayed several days to weeks following injury to permit swelling to subside sufficiently to decrease the risk of wound-healing problems.^[24]^ Percutaneous reduction and fixation should be performed within several days following injury, after which time mobilization of the bony fragments becomes difficult.^[25]^

This study was not without limitations. Due to the retrospective design of the study, patient information and follow-up documentation may lead to underreporting of patient risk factors and postoperative findings. With a relatively low incidence of displaced articular calcaneal fractures, coupled with the low rate of complications, and limited follow-up on some patients, our sample size was likely underpowered to detect differences between fixation methods. PTOA may take longer than 12 months to develop, so we acknowledge the probability of underreporting this complication based on our study design. We also did not obtain postoperative CT scans to more accurately assess reduction quality nor to assess for the development of PTOA. We also did not attempt to measure angular alignment of the calcaneus on the Harris view. Further, we did not include measurements of pain, pain medication intake, or functional outcome scores.^[4,25,41]^ Percutaneous treatment may be effective for tongue type fractures versus those with joint depression due to the ability to manipulate the posterior facet without full exposure. However, our percutaneous group also included joint depression fractures and some patients with more comminution, and the percentage of patients treated percutaneously increased over time, consistent with surgeon learning curve and familiarity.^[21,24]^

## 5 Conclusion

No significant differences in complication rates were found for patients with displaced intra-articular calcaneal fractures treated with ORIF versus percutaneous fixation by a single surgeon and at surgeon discretion. Patients treated with percutaneous fixation had more open fractures and had more comorbidities including nicotine, alcohol and illicit substance abuse. Regardless of fixation technique, patients with open fractures and those with a history of nicotine abuse were more likely to develop complications. Percutaneous reduction and fixation represents an alternative to extensile ORIF in terms of similar early and late complications overall. Percutaneous treatment may provide some improvement in alignment for patients with injury and social risk factors, including abuse of nicotine, alcohol, or recreational drugs, without the major operative risks of an extensile approach.

## Abbreviations

Abbreviations: ORIF = open reduction and internal fixation, PTOA = posttraumatic arthrosis.
